# Blockchain-Enabled iWellChain Framework Integration With the National Medical Referral System: Development and Usability Study

**DOI:** 10.2196/13563

**Published:** 2019-12-04

**Authors:** Yu-Sheng Lo, Cheng-Yi Yang, Hsiung-Fei Chien, Shy-Shin Chang, Chung-Ying Lu, Ray-Jade Chen

**Affiliations:** 1 Graduate Institute of Biomedical Informatics College of Medical Science and Technology Taipei Medical University Taipei Taiwan; 2 Taipei Medical University Hospital Taipei Taiwan; 3 Preventive and Community Medicine Department Taipei Medical University Hospital Taipei Taiwan; 4 Department of Surgery School of Medicine College of Medicine, Taipei Medical University Taipei Taiwan; 5 Department of Family Medicine School of Medicine College of Medicine, Taipei Medical University Taipei Taiwan

**Keywords:** medical referral, electronic referral system, blockchain, decentralized application, electronic medical records, electronic health records, interoperability

## Abstract

**Background:**

Medical referral is the transfer of a patient’s care from one physician to another upon request. This process involves multiple steps that require provider-to-provider and provider-to-patient communication. In Taiwan, the National Health Insurance Administration (NHIA) has implemented a national medical referral (NMR) system, which encourages physicians to refer their patients to different health care facilities to reduce unnecessary hospital visits and the financial stress on the national health insurance. However, the NHIA’s NMR system is a government-based electronic medical referral service, and its referral data access and exchange are limited to authorized clinical professionals using their national health smart cards over the NHIA virtual private network. Therefore, this system lacks scalability and flexibility and cannot establish trusting relationships among patients, family doctors, and specialists.

**Objective:**

To eliminate the existing restrictions of the NHIA’s NMR system, this study developed a scalable, flexible, and blockchain-enabled framework that leverages the NHIA’s NMR referral data to build an alliance-based medical referral service connecting health care facilities.

**Methods:**

We developed a blockchain-enabled framework that can integrate patient referral data from the NHIA’s NMR system with electronic medical record (EMR) and electronic health record (EHR) data of hospitals and community-based clinics to establish an alliance-based medical referral service serving patients, clinics, and hospitals and improve the trust in relationships and transaction security. We also developed a blockchain-enabled personal health record decentralized app (DApp) based on our blockchain-enabled framework for patients to acquire their EMR and EHR data; DApp access logs were collected to assess patients’ behavior and investigate the acceptance of our personal authorization-controlled framework.

**Results:**

The constructed iWellChain Framework was installed in an affiliated teaching hospital and four collaborative clinics. The framework renders all medical referral processes automatic and paperless and facilitates efficient NHIA reimbursements. In addition, the blockchain-enabled iWellChain DApp was distributed for patients to access and control their EMR and EHR data. Analysis of 3 months (September to December 2018) of access logs revealed that patients were highly interested in acquiring health data, especially those of laboratory test reports.

**Conclusions:**

This study is a pioneer of blockchain applications for medical referral services, and the constructed framework and DApp have been applied practically in clinical settings. The iWellChain Framework has the scalability to deploy a blockchain environment effectively for health care facilities; the iWellChain DApp has potential for use with more patient-centered applications to collaborate with the industry and facilitate its adoption.

## Introduction

A medical referral is the transfer of a patient’s care from one physician to another upon request. This process involves multiple steps that require provider-to-provider and provider-to-patient communication [1,2]. A well-designed and robust medical referral system may improve comprehensive health care for all patients by prioritizing those who need it, reducing health inequity, and limiting unnecessary hospital visits as well as the financial burden of health services [3,4]. However, the performance of a medical referral system varies with the level of the health system, efficiency of communication and the referral management process, and costs of different levels of care [5,6].

The Taiwan National Health Insurance (NHI) system, a universal health insurance program implemented in 1995, is characterized by comprehensive population coverage, accessibility, short waiting times, and relatively low costs. However, the weaknesses of this health care system include inconsistent quality of care, weak gatekeeper roles, and increasing financial pressures [7-10]. For the past 20 years, both the average number of hospital visits per person per year and the average number of drug prescriptions per visit have been over two times greater than those in the high-income countries of the Organisation for Economic Cooperation and Development [11]. The immoderate use of health resources and services has become a critical problem for Taiwan. Therefore, leveraging a medical referral system at each level of a health care institution to provide patients with the most appropriate care is one of the most urgent challenges for Taiwan.

In 2017, the Taiwan National Health Insurance Administration (NHIA) launched a national medical referral (NMR) system that encourages physicians to request patient referrals between clinics and hospitals online to reduce unnecessary hospital visits as well as total health care expenditures. Numerous studies have indicated that adopting electronic medical referral systems can improve the referral management process, accessibility of specialty care, and communication between family doctors and specialists, resulting in increased patient and physician satisfaction [3,12,13]. Since May 2018, the NHIA has been provided incentives to encourage physicians at each level of health care facility to issue referrals using the NMR system. Currently, this system supports two operation modes for authorized clinical professionals using their national health smart cards through the NHIA virtual private network (VPN). One mode is online operation through a Web browser interface; the other mode provides an application programming interface (API) to support the integration of the legacy electronic medical record (EMR) systems of hospitals and clinics. In the online operation mode, a physician can use a Web browser to fill in a patient’s referral data when issuing a referral. Subsequently, when the patient visits a health care facility, the referring physician can review the referral data on the Web browser, request that the patient receives treatment, and complete the referral report by replying to the initial health care facility. In the API mode, referral data can be downloaded from or uploaded to the NMR system through batch processing using functions of the API. Therefore, referral data from legacy EMR systems, such as computerized physician order entry (CPOE), can be integrated using the API instead of through the NMR system.

Currently, medical referrals through the NMR system represent a small percentage of the total referrals of hospitals and clinics. Several obstacles limit the use of the existing NMR system. First, physicians are generally too busy to use the online operation mode during a patient consultation; thus, this mode is infeasible. Second, even if referral data can be integrated from the NMR system into the legacy EMR systems, family doctors may hesitate to issue such patient referrals because of concerns about losing the referred patients or corresponding revenue. Third, hospitals may not provide proper patient referral information when they reply to initial facilities because the additional efforts required to collect and prepare the data are extensive. Most importantly, patients who do not have complete medical or health records cannot fully understand their own general health situations. Thus, patients continue seeking health care providers and specialists support independently when they are unsatisfied with the diagnosis or treatment options provided by their initial physicians.

In recent years, blockchain technologies have been characterized as transparent, immutable, and having consensus properties; they are assumed to enable verified, accountable, and pseudoanonymous transactions and as establishing trust and transaction security without any intermediary or middleware [14-18]. These new technologies have been gradually adapted to many industries, especially in health care [19]. According to the distributed ledger model, the blockchain has attracted a lot of attention in health care for its secure, interoperable, and more efficient access to EMR and electronic health record (EHR) data between patients, providers, and relevant participating entities [20-23]. Compared with the existing health information exchange model, patients preferred to adopt the blockchain-enabled applications because of their characteristics of the decentralized data repository, privacy protection, data security, and access control of their EMR and EHR data [24-26]. Thus, blockchain technologies have triggered extensive research interests and applications in finding ways to improve or integrate existing health care workflows and processes [27,28]. Previous studies have proposed to adopt blockchain technologies for facilitating electronic medical referral process [26,29-33]. Some work are conceptual studies [30,31]; the others were being proposed to use the blockchain technologies to construct a flexible architecture of a secured network to improve system efficiency while optimizing security, scalability, and resource allocation. Such examples might be applied in urgent care network, referral network, and primary care physician network [29,33]. Besides, the use of blockchain can extend the existing personal health record (PHR) data management system to combine with event-driven smart contracts to support transactional services (eg, repeat prescription, appointment booking, and referral requests) [26]. Some others have implemented a blockchain-enabled decentralized app (DApp) and framework to address interoperability challenges in health care facilities, and thus, patients can use the DApp to share their clinical information as the basis of decision making for remote support [20,32,34]. Studies about blockchain proposed various technical aspects in the medical referral services; however, health care literature regarding such real-world use cases is lacking in clinical settings [35]. Accordingly, more should be implemented and proven in the health care environment.

According to the Taiwan single-payer NHI system, there are no regulations to force medical referrals for each level of different health care providers. Consequently, patients are free to seek clinical services without referrals, and family doctors can also choose favorable hospitals for patient referrals. Thus, we consider that the establishment of trusting relationships between patients and health care providers is a precondition of EMR and EHR data interoperability [36]. In this study, we aimed to develop a blockchain-enabled framework for building an alliance-based medical referral service connecting health care providers. With this framework, all participants (eg, patients, family doctors, and specialists) who are willing to join the alliance-based medical referral system can create their own blockchain accounts. It is the first step to tighten trust in the relationships for all participants. With the deployment of our framework to health care facilities, it can integrate patient referral data from the NHIA’s NMR system with EMR and EHR data of hospitals and community-based clinics, enhance patients’ EMR and EHR data interoperability across different health care facilities, and make all medical referral processes more efficient. Moreover, we developed a blockchain-enabled PHR DApp based on our framework for patients to acquire their EMR and EHR data. Then, the DApp access logs were collected to assess patients’ behavior and investigate the acceptance of our personal authorization-controlled framework.

## Methods

### Overview

In this study, we adopted the Go Ethereum version 1.7.3-stable [37] to construct the iWellChain Framework, which is a permissioned consortium blockchain with trusted parties to ensure consensus by proof-of-authority. Hence, our framework can limit participants who transact on the blockchain and define users who can serve the network by writing new blocks into the chain. Afterward, we can deploy the framework to hospitals or clinics to build an alliance-based medical referral service. Moreover, regarding the interoperability of EMR and EHR data [38], since 2004, the Taiwan Ministry of Health and Welfare has published EMR and EHR exchange standards based on Clinical Document Architecture, Release 2 (CDA R2) of Health Level 7 (HL7) [39]. Therefore, we followed the HL7 CDA R2 standards when pursuing structural and semantic interoperability of EMR and EHR data. In addition, based on the iWellChain Framework, we developed a DApp, the iWellChain DApp, which can operate on both iOS and Android mobile platforms. Since September 2018, the studied hospital has released five types of EMR and EHR data that patients can acquire through the iWellChain DApp. To extend our understanding of patient-centered interoperability of EMR and EHR data, we analyzed the access logs of the iWellChain DApp to understand patients’ activities during a 3-month study period.

### Settings

For this study, the iWellChain Framework was developed and installed at Taipei Medical University Hospital (TMUH) and four collaborative clinics. TMUH is a teaching hospital with nearly 800 beds and a satisfactory information infrastructure; in addition, it is a certified Health care Information and Management Systems Society hospital using EMR Adoption Model Stage 6 [40]. In this hospital, most medical and health records are stored electronically because the hospital is trying to fully digitize its records. However, when physicians refer patients to other physicians, most patients must acquire paper-based referral forms and related medical documents to be processed manually by clinics and hospitals.

### The iWellChain Framework and Interactions With the National Health Insurance Administration’s National Medical Referral System

**Figure 1:**
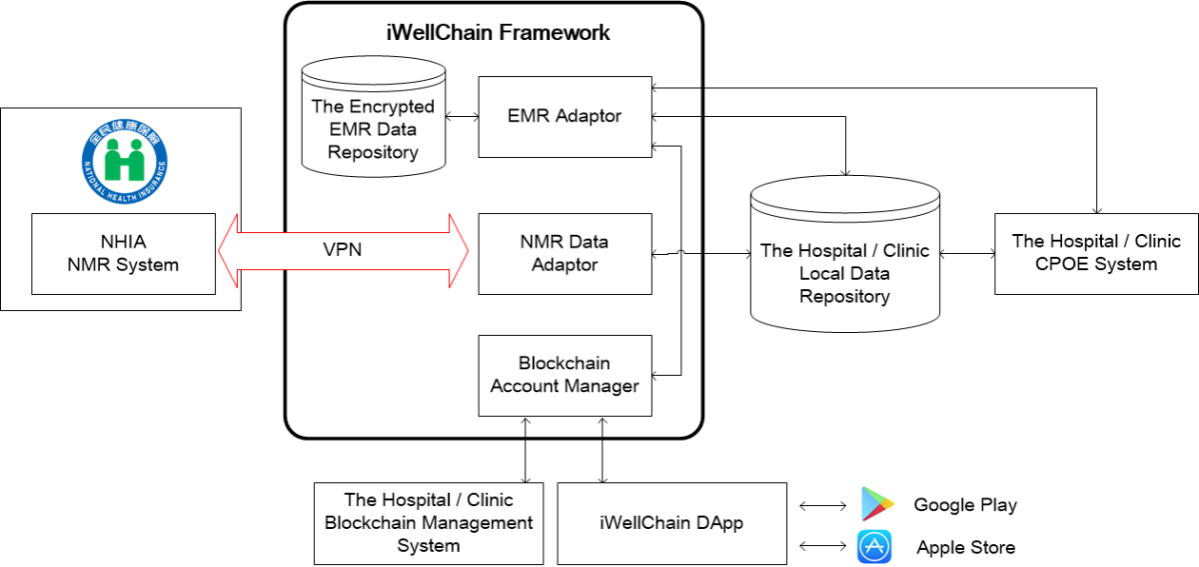
The iWellChain Framework and its interactions with the National Health Insurance Administration’s national medical referral system. CPOE: computerized physician order entry; DApp: decentralized application; EMR: electronic medical record; NHIA: National Health Insurance Administration; NMR: national medical referral; VPN: virtual private network.

The iWellChain Framework and its interactions with the NHIA’s NMR system are presented in Figure 1. The implemented iWellChain Framework comprises the following 4 major components: EMR Adaptor, Encrypted EMR Data Repository, NMR Data Adaptor, and Blockchain Account Manager (BAM).

#### Electronic Medical Record Adaptor

The EMR Adaptor is used to monitor and copy a patient’s EMR and EHR data stored in the local data repository of a hospital or clinic. Moreover, the adaptor can request the patient’s public key from the BAM to encrypt copies of the EMR and EHR data. Subsequently, the adaptor stores these encrypted copies in the Encrypted EMR Data Repository. For example, when a physician uses the CPOE system to complete a patient’s treatment, EMR data are generated. When the patient’s EMR data are ready, the EMR Adaptor verifies the access permissions for each type of EMR data according to the patient’s signed Ethereum-based smart contracts [41], which are provided by the BAM. If permitted, the EMR Adaptor copies the patient’s EMR data and uses the patient’s public key to encrypt these copies. These encrypted copies of the patient’s EMR data are subsequently stored in the Encrypted EMR Data Repository.

The EMR Adaptor also provides a connection that can pose access requests of the CPOE system of a hospital or clinic. For example, when a physician wants to acquire additional EMR or EHR data for a referral patient, the patient can use the iWellChain DApp to determine whom to authorize and select an approved period for the physician to access the EMR and EHR data. Accordingly, the selected EMR and EHR data are decrypted by the patient’s private key. With the patient’s permission, the EMR Adaptor can request the physician’s public key from the BAM to encrypt the copies of the patient’s selected EMR and EHR data. Subsequently, the EMR Adaptor stores these encrypted copies in the Encrypted EMR Data Repository. Afterward, the physician can use his or her private key to open the selected patient EMR and EHR data through the CPOE system of a hospital or clinic until the time at which access to the patient’s EMR and EHR data expires.

#### Encrypted Electronic Medical Record Data Repository

The Encrypted EMR Data Repository is implemented using the InterPlanetary File System (IPFS) [42,43] to store encrypted copies of EMR and EHR data. The IPFS provides a high-throughput content-addressed block storage model with content-addressed hyperlinks, which enable completely distributed applications. When the Encrypted EMR Data Repository receives copies of a patient’s encrypted EMR and EHR data, a unique hash code is generated for each copy, and it corresponds to an accessible hyperlink. The hash code is registered on the BAM for the iWellChain DApp to access and acquire further data.

#### National Medical Referral Data Adaptor

The NMR Data Adaptor is used in cooperation with the NMR’s API functions to process patient referral data and reports obtained in batch mode from the NHIA’s NMR system over the VPN. This adaptor can retrieve referral data through batch download from the NHIA’s NMR system and store these data in the local data repository of a hospital or clinic. When patient referral reports are completed and the physician must reply to the initial health care facility, the NMR Data Adaptor can access these reports from the local data repository through batch upload to the NMR system.

#### Blockchain Account Manager

The BAM module was designed to access Ethereum blockchain ledgers [44], and it provides the registration function of the iWellChain DApp. The BAM was installed at the information desk of TMUH with the Ethereum node client software [45] to access the full ledgers of Ethereum. When a patient downloads and installs the iWellChain DApp from Google Play or the Apple Store, a wallet address (a patient’s identification [ID] in the Ethereum World) is generated. Patients can register an account from the iWellChain DApp to the BAM using their national ID (a unique personal ID in Taiwan) and medical record number (unique patient ID at TMUH). Accordingly, the registration information and the patient’s ID mapping table are deposited into and managed by the BAM. Patients can grant access to their EMR and EHR data to whomever they choose through the iWellChain DApp; these permissions are recorded in the ledger.

### Log Data of the iWellChain Decentralized App

To investigate patients’ use of the iWellChain DApp, an access log was created, and information on their actions through the iWellChain DApp was collected. We added Google Analytics for Firebase [46] to the iWellChain DApp to capture patients’ click data. Once the data are captured, the access log can be linked to Google BigQuery for analysis and reporting [47]. Besides, all physician access logs are stored in the event logs of a smart contract through the iWellChain Framework. When a physician acquires a patient’s EMR or EHR data via the CPOE system, the iWellChain Framework signs access logs in the event logs of a smart contract using the physician’s private key. The Joint Institutional Review Board of Taipei Medical University and TMUH approved this study.

## Results

### Workflow for Referral Between Clinics and Hospitals Based on the National Health Insurance Administration’s National Medical Referral System and the iWellChain Framework

**Figure 2:**
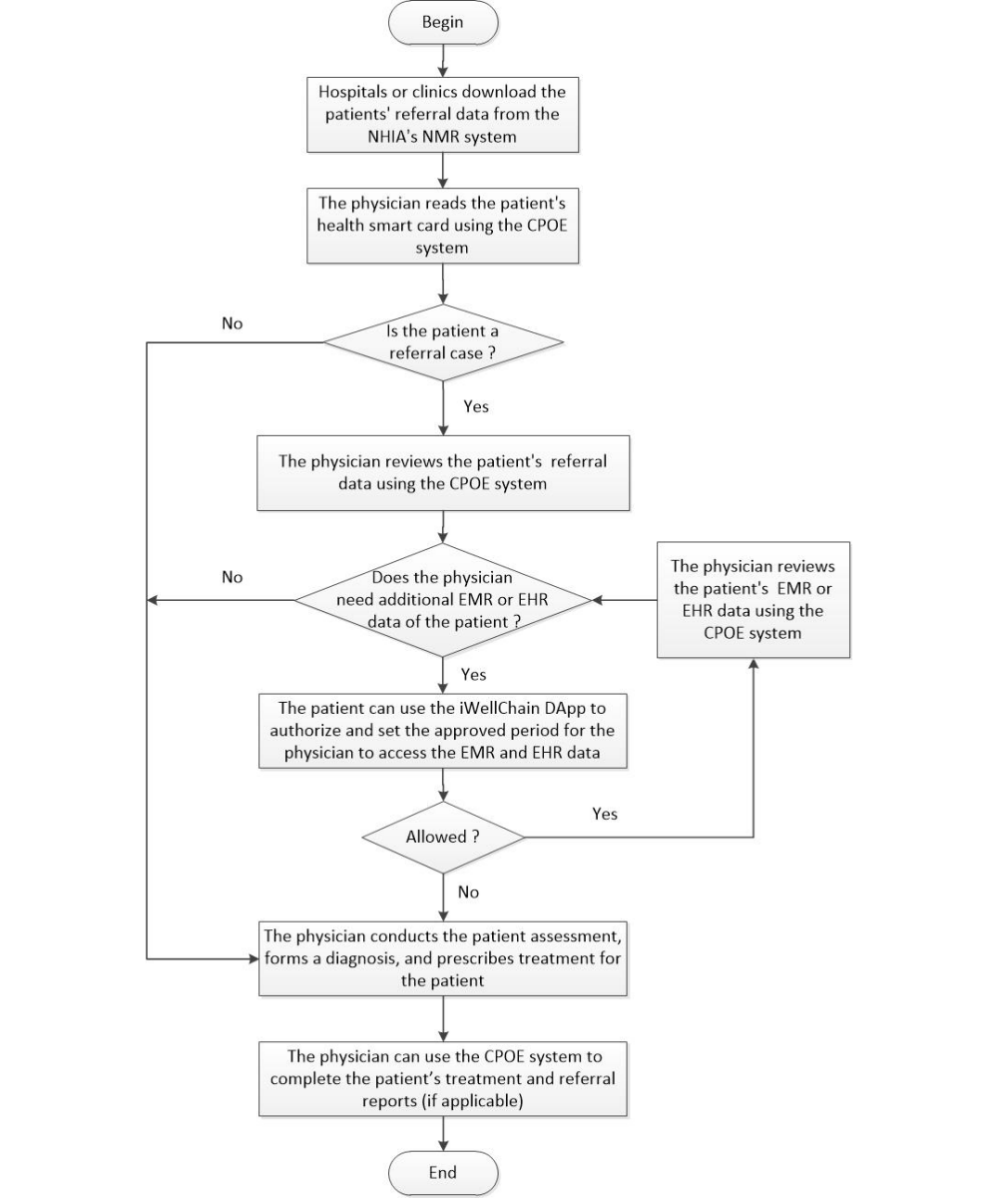
Workflow of the alliance-based medical referral service using the iWellChain Framework. CPOE: computerized physician order entry; DApp: decentralized application; EHR: electronic health record; EMR: electronic medical record; NHIA: National Health Insurance Administration; NMR: national medical referral.

Patients wishing to access and control their EMR and EHR data must create a blockchain account at a health care facility and sign and submit the appropriate smart contracts to the health care facility. The patients can subsequently download the iWellChain DApp and use it to access their EMR and EHR data. In addition, family doctors and specialists willing to join TMUH’s alliance-based medical referral system can create their own blockchain accounts. The objective is to enhance EMR and EHR data sharing among facilities, with patients’ permission. The novel workflow of patient referral between clinics and hospitals with the iWellChain Framework is described in Figure 2.

**Figure 3:**
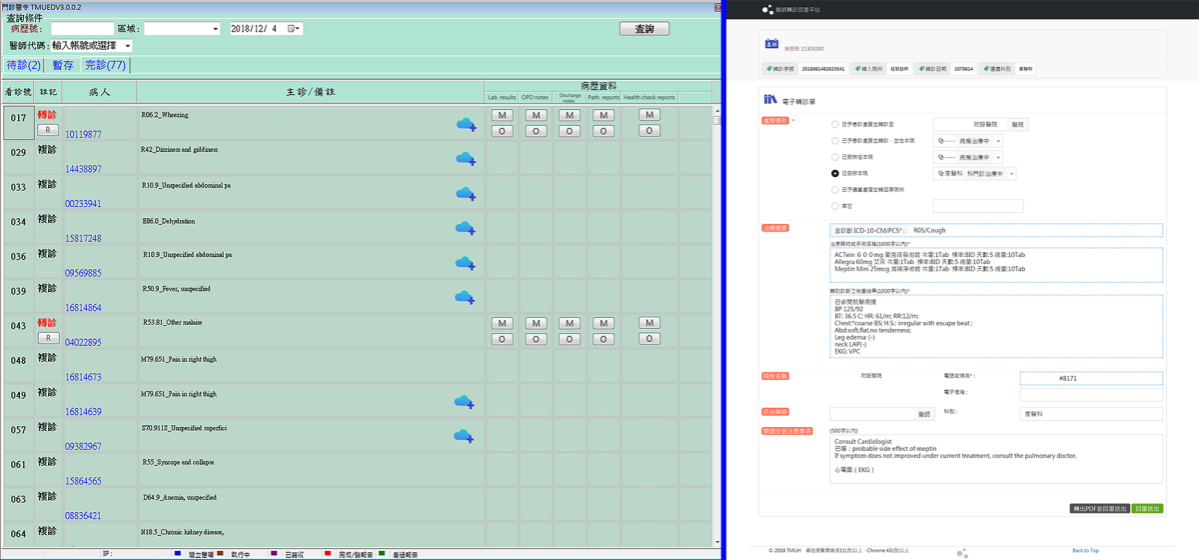
Screenshot of patient list screen showing the R icon that appears when an outpatient has been referred. The screen presents information including the patient list (left panel), referral cases with R icons (left part of the left panel), and display area for referral data from the National Health Insurance Administration’s national medical referral system (right panel).

Figure 2 illustrates the workflow of the alliance-based medical referral service used by patients, clinics, and hospitals using the iWellChain Framework. First, when visiting a health care facility, the patient inserts his or her health smart card into a card reader to initiate the clinical encounter. The CPOE system reads the basic information on the card, including name, sex, and national ID. Patient information is compared with the downloaded referral data to determine whether the patient has been referred. If the patient has been referred (denoted by the *R* icon in Figure 3, left part of the left panel), the physician can click on the *R* icon to review the patient’s referral data (Figure 3, right panel). The indexes of the patient’s EMR and EHR data (Figure 3, right part of the left panel) can be accessed, including laboratory test reports, outpatient notes, discharge notes, pathology reports, and health check reports. However, if the patient is not a referral case, the physician conducts the usual patient assessment, forms a diagnosis, and prescribes treatment for the patient.

**Figure 4:**
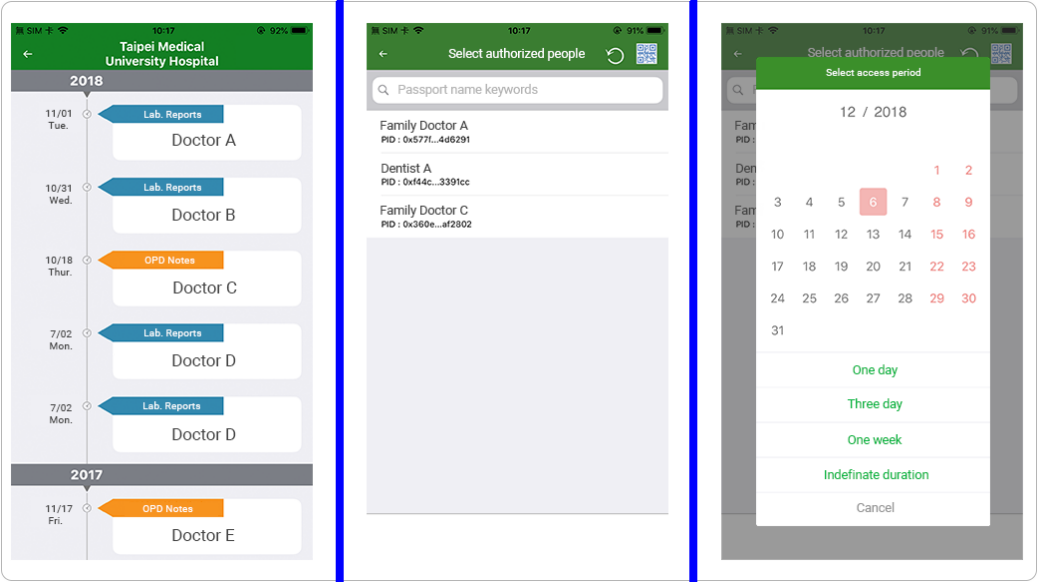
Screenshot of the patient iWellChain decentralized application screen. The screen presents information including a timeline of the patient’s electronic medical record and electronic health record data (left panel), an authorization list (middle panel), and the approved access time for the selected electronic medical record and electronic health record data (right panel). Lab.: laboratory; OPD: outpatient department.

If requesting additional EMR or EHR data for a referral patient, a physician clicks on the *M* icon (Figure 3, right part of the left panel) to send a push notification to the patient’s mobile phone. As the push notification is received by the patient via the iWellChain DApp, the patient can use the DApp to select specific EMR or EHR data (Figure 4, left panel), determine whom (ie, physicians or health care workers) to authorize (Figure 4, middle panel), and select the approved period for the physician to access the EMR and EHR data (Figure 4, right panel). Subsequently, the physician can click the *O* icon (Figure 3, right part of the left panel) to access the EMR and EHR data provided by the initial facility via the CPOE system. If the physician’s access to the patient’s EMR and EHR data is not permitted or has expired, a notification window will appear when the physician clicks on the *O* icon. In this case, the physician can revert to using the CPOE system to assess, diagnose, and treat the patient. After treatment, the physician can use the CPOE system to complete the patient’s treatment and referral reports (if applicable), generate the patient’s EMR data, and store these data in the local data repository of the clinic or hospital. The iWellChain Framework can then assist in subsequent tasks. For example, patients can obtain their EMR data in real time using the iWellChain DApp, and referral reports can be uploaded to the NHIA’s NMR system to complete the referral process for NHIA reimbursement claims.

### Access Log Data Analysis of the iWellChain Decentralized App

The iWellChain Framework and iWellChain DApp were launched on September 16, 2018. At this time, TMUH informed the public about the possible application of blockchain technologies to support medical referral services and the use of the iWellChain DApp to access patient EMR and EHR data [48,49]. Accordingly, when patients visited TMUH to seek clinical services, the hospital’s staff, which mainly included workers from the health check department, community medicine department, and customer service department, suggested these patients create a blockchain account. The health check department encouraged customers who received high-end health check services (>US $1000) to create blockchain accounts to deliver the health check reports rapidly. On average, the number of high-end health check customers was approximately 150 to 250 per month. The community medicine department encouraged patients who were referred via the NHIA’s NMR system to create blockchain accounts to facilitate referral report completion and NHIA insurance reimbursement. On average, the number of such referral patients was approximately 250 to 350 per month. Moreover, patients who wished to create the blockchain accounts were free to seek help from the information desk of the customer service department. The information desk staff helped patients install the iWellChain DApp and offered assistance through technical tutorials. In the initial stages, TMUH released only five types of EMR and EHR data for the patients to retrieve: laboratory test reports, outpatient department (OPD) notes, discharge notes, pathology reports, and health check reports. We collected the blockchain account data from the 3 months following implementation (September 16, 2018, to December 15, 2018) to explore the patients’ application of their accounts. We also analyzed the iWellChain DApp access logs for these accounts to determine which EMR and EHR data patients required the most.

Over the 3-month period, the total numbers of high-end health checkup customers, referral patients through the NHIA’s NMR system, and individual apps were 581, 912, and 118, respectively. Table 1 reveals that over this 3-month period, 392 users registered iWellChain DApp accounts; the health check department, community medicine department, and individual application channels were used by 175 (175/392, 44.6%), 99 (99/392, 25.3%), and 118 users (118/392, 30.1%), respectively. The highest proportion of users who requested an account was from the health check department channel. This can be explained by the fact that the health check department promoted the iWellChain DApp service as a new customer service capable of delivering health check reports to customers within 1 day. The service also enhances the personal security and privacy of patients by allowing them to access health check reports using a secured blockchain network instead of a paper-based health document. Moreover, computed tomography and magnetic resonance imaging images from a compact disc often take weeks to be delivered. Per our understanding, most users who applied independently were young and thus might be attracted to or interested in blockchain technologies. They wanted to understand how blockchain technologies can be applied to health care. Most users referred by the community medicine department were from family groups, including older patients who were attracted by the hospital’s newsletter. The caregivers of older patients wanted to understand the patients’ general health to avoid returning to the hospital and to reduce the time spent accompanying and waiting with patients and associated medical costs.

Over this 3-month period, as Table 2 indicates, the total numbers of laboratory test reports, OPD notes, discharge notes, pathology reports, and health check reports were 406, 1358, 2, 134, and 175, respectively. Table 2 displays the total user access logs of the iWellChain DApp over this 3-month period. Overall, there were 4540 (80.09%), 721 (12.85%), 7 (0.12%), 4 (0.07%), and 340 (6.06%) clicks on the laboratory test reports, OPD notes, discharge notes, pathology reports, and health check reports, respectively. Laboratory test reports being the most frequently accessed is likely explained by the fact that most patients are focused on the value of laboratory tests, especially when they have been examined in multiple hospitals or when their tests results require constant monitoring of the condition. Some patients may even doubt the accuracy of their laboratory tests. The high access to OPD notes and health check reports we observed corresponds to patients or their families wanting to understand the patients’ long-term medication history and health status. The lowest access to discharge notes and pathology reports is related to the fact that these types of medical documents are long text descriptions; therefore, patients have difficulty understanding them. In addition, patients often applied for these documents in paper form before discharge because they needed the documents to claim private insurance paybacks.

**Table 1:** Descriptive statistics of blockchain accounts registered through various application channels.

Time period	Total users, n (%)	Health check department			Community medicine department				Individual application (total registered users, n [% of total users])	
		Total visits^a^	Total registered users, n (% of total visits)	Total registered users/total users (%)		Total visits^b^	Total registered users, n (% of total visits)	Total registered users/total users (%)		
Entire period	392 (100)	581	175 (30.1)	44.6		912	99 (10.9)	25.3		118 (30.1)
Period I^c^	231 (100)	158	88 (55.7)	38.1		325	54 (16.6)	23.4		89 (38.5)
Period II^d^	56 (100)	215	8 (3.7)	14.3		314	25 (8.0)	44.6		23 (41.1)
Period III^e^	105 (100)	208	79 (38.0)	75.2		273	20 (7.3)	19.1		6 (5.7)

^a^Total number of high-end health check visits.

^b^Total number of referral patients via the National Health Insurance Administration’s national medical referral system.

^c^Period I: September 16, 2018, through October 15, 2018.

^d^Period II: October 16, 2018, through November 15, 2018.

^e^Period III: November 16, 2018, through December 15, 2018.

**Table 2:** Descriptive statistics of user access logs to iWellChain decentralized app.

Time period	Total clicks, n (%)	Laboratory tests reports		Outpatient department notes			Discharge notes			Pathology reports		Health check reports	
		Total reports^a^, n (%)	Total clicks, n (%)		Total notes^b^ (%)	Total clicks, n (%)		Total notes^c^, n (%)	Total clicks, n (%)	Total reports^d^, n (%)	Total clicks, n (%)	Total reports^e^, n (%)	Total clicks, n (%)
Entire period	5612 (100)	406 (100)	4540 (80.90)		1358 (100)	721 (12.85)		2 (100)	7 (0.12)	134 (100)	4 (0.07)	175 (100)	340 (6.06)
Period I^f^	2878 (100)	116 (28.6)	2302 (79.99)		281 (20.69)	362 (12.58)		1 (50)	2 (0.07)	19 (14.2)	1 (0.03)	88 (50.3)	211 (7.33)
Period II^g^	906 (100)	82 (20.2)	789 (87.1)		530 (39.03)	88 (9.7)		0 (0)	2 (0.2)	31 (23.1)	0 (0)	8 (4.6)	27 (3.0)
Period III^h^	1828 (100)	208 (51.2)	1,449 (79.27)		547 (40.28)	271 (14.82)		1 (50)	3 (0.16)	84 (62.7)	3 (0.16)	79 (45.1)	102 (5.58)

^a^Total number of laboratory test reports.

^b^Total number of outpatient department notes.

^c^Total number of discharge notes.

^d^Total number of pathology reports.

^e^Total number of health check reports.

^f^Period I: September 16, 2018, through October 15, 2018.

^g^Period II: October 16, 2018, through November 15, 2018.

^h^Period III: November 16, 2018, through December 15, 2018.

## Discussion

### Principal Findings

In this study, we developed the iWellChain Framework to access and integrate patients’ referral data, including EMR and EHR data from hospitals and clinics, from the NHIA’s NMR system. We used blockchain technology to render the data interoperable and practical. Our framework has the scalability to deploy a blockchain environment effectively for health care settings. It is an independent architecture that does not affect the legacy EMR systems of hospitals or clinics. Although some literature has proposed adopting blockchain technology to support medical referral processes, researchers have focused on improving the system efficiency by optimizing security and scalability but have not applied the technology practically in clinical settings. On the basis of what we learned, this study is the pioneer of blockchain applications for medical referral services, and the constructed framework and DApp have been applied practically in clinical settings [49]. Using the batch processing mode of the NHIA’s NMR system, our framework can make all medical referral processes automatic, paperless, and thus more efficient; in addition, it can assist health care facilities with NHIA reimbursements.

According to Taiwan’s NHIA policies announced in 2017, the number of outpatient visits to hospitals must decrease by 2% per year for a total decrease of 10% over 5 years. The NHIA aims to curb the overuse of health resources and discourage patients with minor ailments from seeking treatment at major hospitals. In other words, if a hospital has a higher proportion of outpatients with minor ailments, its NHI reimbursements will be reduced. Therefore, hospitals are encouraging patients to avoid unnecessary outpatient visits to conform with the regulations. Thus, trusting relationships and patient-centered data interoperability between patients and health care facilities are becoming crucial [20]. The NHIA’s NMR system provides a government-based electronic medical referral service. However, its access and exchange of referral data are limited only to authorized clinical professionals. Thus, the system lacks flexibility and is not suitable to establish an alliance-based referral service connecting patients, community-based clinics, and hospitals. Accordingly, we intend to deploy our iWellChain Framework gradually in cooperative clinics to make improvements in data interoperability and tighten trust in the relationships among patients, family doctors, and specialists. Although more than 100 community clinics collaborated with the study hospital, we deployed the iWellChain Framework at only four clinics in the initial stage (September 2018 through January 2019). Therefore, the participants have been limited, and the total transaction volume of EMR and EHR data has not become a bottleneck. However, we consider that blockchain scalability is a major challenge, especially in relation to the volume of health data involved. In this study, we tested the transaction capacity of the iWellChain Framework. Regarding Ethereum, the average transaction time to mine a block is approximately 15 seconds; however, the time cost varies significantly with the network environment. Currently, the average transaction execution time for the iWellChain Framework is 5 to 7 seconds, and its performance will increase gradually because of the novel consensus mechanism. Therefore, blockchain scalability must be further observed and improved considerably. In the next stage of iWellChain Framework implementation, we will gradually extend the alliance-based referral service and use blockchain technologies to address common challenges in health care, such as the physician referral process and data interoperability among health care facilities and patients.

Conventionally, the interoperability of EMR and EHR data in health care has mostly been the ability of health care providers to access patients’ relevant clinical data to provide a high-quality of care [50,51]. This interoperability is, therefore, institution-centered and motivated by financial incentives or regulatory pressure [9,52,53]. However, the current trend of interoperability in health care is to transform this institution-centered interoperability into patient-centered interoperability [36]. Blockchain technology can be used to promote this change to patient centricity, which concerns both data sharing and patients’ privacy and security [35,41,54]. The iWellChain DApp is a blockchain-enabled PHR tool for patients to acquire their EMR and EHR data securely and electronically. It empowers patients to control these data as health data assets and selectively share or sell them [27,55]. As described in the Results section, the high-end health checkup customers (n=581), patients referred via the NHIA’s NMR system (n=912), and individual applications (n=118) represented 1611 potential users; however, in the initial stage (3 months), only 392 users (24.33%) created blockchain accounts (Table 1). According to our observations, although most older patients would like to install the iWellChain DApp to acquire their EMR or EHR data, most of their cell phones are not sufficiently modern to install it. Another observation is that patients were especially interested (Table 2) in acquiring their EMR and EHR data for laboratory test reports. In a clinical scenario, through the TMUH alliance-based referral network, patients who previously visited TMUH for OPD services can use the iWellChain DApp to check laboratory test results and authorize these clinical data to be shared with their cooperative family doctors. If necessary, patients can first consult family doctors, instead of going to hospitals, especially for those with chronic conditions or long-term illnesses. Accordingly, blockchain technology may reduce costs by increasing speed and efficiency in the management of health data. We analyzed use of the app from the health check department channel by comparing clicks on EMR and EHR data. Although the total number of health check reports was low (n=175), such users presented greater interest in health check reports, as evidenced by the total clicks (n=340) using the iWellChain DApp (Table 2). We believe that the number of blockchain users who care about their health data will increase gradually.

Using a blockchain-enabled approach to design an electronic medical referral service with patient-centered principles was our first exciting experience in health care. We may be able to achieve patient-centered interoperability of practical EMR and EHR data. As with numerous other new frontiers in information technology, we should be ambitious but should take measured early steps with blockchain. Accordingly, with the increased adoption of blockchain technology, more meaningful patient-centered applications will require the involvement of multiple stakeholders, including health providers, health participants, and individual governments, and have the potential to revolutionize health care.

### Conclusions

In this study, we constructed the blockchain-enabled iWellChain Framework for integrating patients’ referral data from the NHIA’s NMR system with the EMR and EHR data of hospitals and clinics. This framework can render all medical referral processes automatic, paperless, and efficient, facilitating NHIA reimbursements. In addition, the iWellChain Framework possesses the scalability to deploy a blockchain environment effectively for hospitals and community-based clinics. The framework assists in the establishment of an alliance-based medical referral service to promote trusting relationships and transaction security among patients, family doctors, and specialists. We also developed the iWellChain DApp, which is a blockchain-enabled PHR tool, focused on patient-centered interoperability, that allows patients to access and control their EMR and EHR data securely and electronically. Crucially, we observed that in the initial stage, patients were highly interested in acquiring their health data using the iWellChain DApp, especially data from laboratory test reports. Future research should further explore patient-centered interoperability and involve multiple stakeholders, including health care providers, health participants, and governments.

## Abbreviations

API: application programming interface
BAM: Blockchain Account Manager
CDA R2: Clinical Document Architecture, Release 2
CPOE: computerized physician order entry
DApp: decentralized application
EHR: electronic health record
EMR: electronic medical record
HL7: Health Level 7
IPFS: InterPlanetary File System
NHI: National Health Insurance
NHIA: National Health Insurance Administration
NMR: national medical referral
OPD: outpatient department
PHR: personal health record
TMUH: Taipei Medical University Hospital
VPN: virtual private network
